# COVID-19 vaccine uptake, hesitancy and clinical effects on patients with Takayasu’s arteritis: A web-based questionnaire survey from a large cohort

**DOI:** 10.3389/fimmu.2023.1030810

**Published:** 2023-02-09

**Authors:** Xiufang Kong, Xiaojuan Dai, Lingying Ma, Jinghua Wang, Ying Sun, Lindi Jiang

**Affiliations:** ^1^ Department of Rheumatology, Zhongshan Hospital, Fudan University, Shanghai, China; ^2^ Center of Clinical Epidemiology and Evidence-based Medicine, Fudan University, Shanghai, China

**Keywords:** takayasu arteritis, COVID-19, vaccination, clinical effects, disease activation

## Abstract

**Objective:**

This study aimed to investigate the Coronavirus disease 2019 (COVID-19) vaccination rate, reasons for vaccine hesitancy and clinical effects on patients with Takayasu’s arteritis (TAK).

**Methods:**

A web-based survey was administered to a TAK cohort established by the Department of Rheumatology, Zhongshan Hospital through WeChat in April, 2022. Responses from a total of 302 patients were received. The Sinovac or Sinopharm inactivated vaccination rate, side effects, and vaccine hesitancy reasons were analyzed. In addition, disease flare, new disease onset, and changes of immune-related parameters after vaccination were analyzed in vaccinated patients.

**Results:**

Among 302 patients, 93 (30.79%) received the inactivated COVID-19 vaccination. Among the 209 unvaccinated patients, the most common reason for hesitancy were concern about side effects (136, 65.07%). Vaccinated patients had a longer disease duration (p = 0.08) and lower use of biologic agents (p < 0.001); 16 (17.20%) of the 93 vaccinated patients developed side effects, and most of them were mild; 8 (8.60%) developed disease flares or new-onset disease 12-128 days post-vaccination and 2 (2.15%) developed serious adverse effects (vision defect and cranial infarction). Immune-related parameters of 17 patients indicated decreases in IgA and IgM after vaccination (p < 0.05). Eighteen (19.35%) of the 93 vaccinated patients were diagnosed post-vaccination.These patients had a significantly higher percentage of CD19^+^ B cells at disease onset (p < 0.05) than the unvaccinated patients diagnosed at the same time.

**Conclusion:**

The vaccination rate was low in TAK, which was mainly caused by concerns about negative effects of vaccination on their disease. An acceptable safety profile was observed in vaccinated patients. The risk of disease flare associated with COVID-19 vaccination warrants further investigation.

## Introduction

Since the outbreak of Coronavirus disease 2019 (COVID-19) in December 2019, COVID-19 has become a global pandemic and a pressing public health concern worldwide. Vaccination is an effective way to curb its transmission and protect populations from infection. There are currently several types of COVID-19 vaccinations, including an inactivated vaccine, messenger RNA (mRNA) vaccine, vector vaccine and protein subunit vaccine (1); the inactivated vaccination is the main type used in China. Vaccinations have been reported to protect over 60% of adults and children age 5 years and older from becoming sick or severely ill with COVID-19 (2). To date, approximately 66.7% of the world’s population has received at least one dose of the COVID-19 vaccination (3).

However, many populations with underlying illnesses, especially those with autoimmune diseases, immune deficiency diseases, and cancer are unwilling to be vaccinated due to multiple uncertainties (4). Takayasu’s arteritis (TAK) is a rheumatic disease involving the aorta and its main branches, which predominantly occurs in females of childbearing age (≤ 50 years) (5). Abnormal activation of immune response plays a pathogenic role in the pathogenesis of TAK, and most patients are under treatment with glucocorticoids combined with immunosuppressive agents (6). Patients with rheumatic disorders have a relatively higher mortality rate than the general population and patients with other chronic diseases, such as diabetes, chronic kidney disease and cardiovascular disease (7). However, social, family or personal factors may influence their decision to get vaccinated.

So far, specific data on the uptake rate of the COVID-19 vaccination among patients with TAK and possible clinical effects of the vaccination on the severity of their disease have not been reported. Therefore, this study investigated the vaccination status, reasons for vaccine hesitancy and potential clinical effects of the COVID-19 vaccination on a large cohort of patients with TAK.

## Methods

### Patients

This study was conducted with patients in the East China Takayasu Arteritis cohort in China, which was established on January 1st, 2009 by the Department of Rheumatology of Zhongshan Hospital in Shanghai, China. Patients in the cohort were mainly from Shanghai and the surrounding areas in the eastern region of China. All of them had been admitted to the hospital’s Department of Rheumatology and were diagnosed with TAK in accordance with the classification criteria of the 1990 American College of Rheumatology (8). All the patients were regularly followed up and physically assessed at each visit. During the pandemic situation, patients could be followed up at local centers with rheumatic department and follow-up data including physical assessment were collected. Patients’ clinical data were recorded in an electronic database by one designated person, who was also responsible for maintaining contact with patients *via* WeChat. There are 465 patients with TAK on our WeChat-contact list so far.

The study was approved by the Institutional Review Board of Zhongshan Hospital, Fudan University, China (approval number: B-2016-168(2)R) and it conforms to the provisions of the Declaration of Helsinki. Consents have been obtained from all the patients before participating in this survey.

### Web-based survey of patients regarding COVID-19 vaccination

The study design was shown in **Supplementary Figure S1**. An electronic questionnaire that was distributed to 465 patients through WeChat in April, 2022. This survey was not anonymous. The questionnaire surveyed three major areas: demographics, vaccination and clinical status. The vaccination-related questions included COVID-19 vaccination status, dosage, injection date, side effects post-vaccination and reasons for not being vaccinated. Regarding with side effects, serious adverse events included deaths or events that were life-threatening, required inpatient hospitalization or resulted in persistent damage or significant disability. Clinical-related questions consisted of effect(s) of the vaccination on disease status (any worsening of TAK-related signs or symptoms), and changes in medication dosage or treatment regimen after vaccination. The government of China has provided free inactivated vaccines to citizens since January 2021. The vaccinations mainly include Sinovac COVID-19 vaccine (Sinovac Life Sciences, Beijing, China) and Sinopharm China Biological Vaccine (Beijing Institute of Biological Products, Beijing, China).

### Clinical data collection and analysis

A total of 302 patients responded to the questionnaire. Correspondingly, their clinical data included disease duration, disease activity, imaging types, organ involvement and treatment regimen before the vaccination rollout were collected from out database. Disease activity was assessed based on the criteria of the National Institutes of Health (≥ 2 points) (9). Vascular imaging types were classified in accordance with the 1996 Numano classification (10). Organ involvement included organ failure, organ atrophy or a severe ischemic event of a specific organ due to severe stenosis or occlusion of a corresponding artery. These conditions included cerebral infarction, renal failure, renal atrophy, pulmonary infarction, cardiac infarction and heart failure. Clinical characteristics were compared between patients with and without a vaccination, and vaccination rates among patients were analyzed by clinical features such as imaging types, organ involvement and treatment regimens.

Lab test results of immune-related parameters were collected from 17 patients within six months prior to the first dose of the COVID-19 vaccination and three months after the second dose. These parameters included the erythrocyte sedimentation rate (ESR), C-reactive protein (CRP), immunoglobulin G (IgG), IgM, IgA, IgE, interleukin 1β (IL-1β), IL-6, IL-8, IL-2 receptor (IL-2R), IL-10, tumor necrosis factor α (TNF-α), complement 3 (C3), C4, CH50, and the percentages of the different immune cells, such as the CD19^+^ B, CD3^+^ T, CD4^+^ T, CD8^+^ T and natural killer (CD56^+^) cells. Changes in these parameters after vaccination were analyzed to evaluate the potential effects of COVID-19 vaccination on the patient’s immune function.

Eighteen patients were diagnosed post-vaccination based on the injection date from May 2021 to April 2022. Data on the duration between the TAK diagnosis and the last vaccination, onset of signs or symptoms, ESR and CRP were also collected. During the same period, 21 unvaccinated patients were diagnosed with TAK. Their clinical features, especially their onset of signs and symptoms were compared to examine the effects of the COVID-19 vaccination on the onset of TAK.

### Statistical analysis

Categorical data are expressed as numbers and percentages; continuous variables that are normally distributed are expressed as mean ± standard deviation (SD) and continuous variables that are not normally distributed are expressed as median and interquartile range. The independent samples *t*-test, χ2 test or Fisher’s exact test was used to compare the demographic and clinical characteristics between patients in the vaccinated and unvaccinated groups. SPSS 25.0 (IBM, Armonk, NY, USA) and Prism 9.1.0 (GraphPad, La Jolla, CA, USA) were used for the statistical analyses.

## Results

### Patients’ characteristics

No significant differences were found in the demographic or clinical features between the responders (n = 302) and non-responders to the vaccination survey (n = 163) (**Supplemental Table S1**). The demographic and clinical characteristics of the 302 patients who answered the questionnaire are shown in **Table 1**. Their mean age was 33.69 ± 13.32 years, their (female: male) gender ratio was 264: 38, and their disease duration was approximately 35 (16.25, 58.00) months. Imaging type V (132, 43.71%) was most common among these patients, and 92 (30.46%) patients had TAK-associated organ involvement. The major treatment for patients at the vaccination rollout included glucocorticoids (GCs) combined with biologic disease-modifying anti-rheumatic drugs (DMARDs) (101, 33.44%), GCs combined with conventional DMARDs (170, 56.29%), GCs alone (13, 4.30%) and no treatment (18, 5.96%).

**Table 1:** Characteristics of the vaccinated and unvaccinated groups.

Characteristics	Total (N=302)	Unvaccinated group (n=209)	Vaccinated group (n=93)	p
**Age at diagnosis (mean ± SD, years)**	30.59 ± 10.30	30.34 ± 9.96	31.15 ± 11.06	0.53
**Age at vaccination rollout (mean ± SD, years)**	33.69 ± 13.32	33.59 ± 14.38	33.91 ± 10.68	0.84
**Sex (female: male, ratio)**	264: 38	179:30	85:8	0.19
**Disease duration (median, IQR, months)**	35 (16.25, 58.00)	31(16.00, 55.00)	41.5 (20.75, 64.25)	**0.08**
**Diagnosed since the year 2020, n (%)**	118 (39.07)	90 (43.06)	28 (30.11)	**0.04**
**Been active in the past year, n (%)**	72 (23.84)	54 (25.84)	18 (19.35)	0.24
Imaging type, n (%)				0.24
I	85 (28.14)	58 (27.75)	27 (29.03)	
IIA	12 (3.97)	11 (5.26)	1 (1.08)	
IIB	43 (14.24)	27 (12.92)	16 (17.20)	
III	13 (4.30)	7 (3.35)	6 (6.45)	
IV	17 (5.63)	10 (4.78)	7 (7.53)	
V	132 (43.71)	96 (45.93)	36 (38.71)	
TAK-associated organ involvement, n (%)	92 (30.46)	65 (31.10)	27 (29.03)	0.74
Cerebral infarction	28 (9.27)	21 (10.05)	7 (7.53)	
Pulmonary infarction	4 (1.32)	3 (1.44)	1 (1.08)	
Heart failure or cardiac infarction	25 (8.28)	15 (7.18)	10 (10.75)	
Renal dysfunction or renal atrophy	35 (11.59)	26 (12.44)	9 (9.68)	
Major treatment				<0.001†
GCs and Biologic DMARDs, n (%)	101 (33.44)	84 (40.19)	17 (18.28)	
TOF	40 (13.26)	30 (14.35)	10 (10.75)	
TCZ	33 (10.93)	28 (13.40)	5 (5.37)	
ADA	21 (6.95)	20 (9.57)	1 (1.08)	
IL17Ab	7 (2.32)	6 (2.87)	1 (1.08)	
GCs and Conventional DMARDs, n (%)	170 (56.29)	117 (55.98)	53 (56.99)	
LEF	85 (28.15)	67 (32.06)	18 (19.35)	
MTX	20 (6.62)	13 (6.22)	7 (7.53)	
MMF	15 (4.97)	8 (3.83)	7 (7.53)	
AZA	12 (3.97)	7 (3.35)	5 (5.38)	
CTX	6 (1.99)	3 (1.44)	3 (3.22)	
HCQ	14 (4.64)	10 (4.78)	4 (4.30)	
Others*	18 (5.96)	9 (4.31)	9 (9.68)	
**Single GCs**	13 (4.30)	8 (3.83)	5 (5.38)	0.54
**No treatment**	18 (5.96)	/	18 (19.35)	

SD, standard deviation; IQR, interquartile range; ADA, adalimumab; TCZ, Tocilizumab; AZA, Azathioprine; TOF, tofacitinib; LEF, leflunomide; MMF, mycophenolate mofetil; IL17Ab, Secukinumab; DMARDs, disease-modifying anti-rheumatic drugs; MTX, methotrexate; CTX, cyclophosphamide; HCQ, hydroxychloroquine; GCs, glucocorticoids; *Others: curcumin, Tacrolimus and sirolimus; † indicates comparisons of treatment with biologic and conventional DMARDs, GC alone and no treatment between the vaccinated and unvaccinated groups.

### COVID-19 vaccination status of patients with TAK and their reasons for not being vaccinated

**Figure 1:**
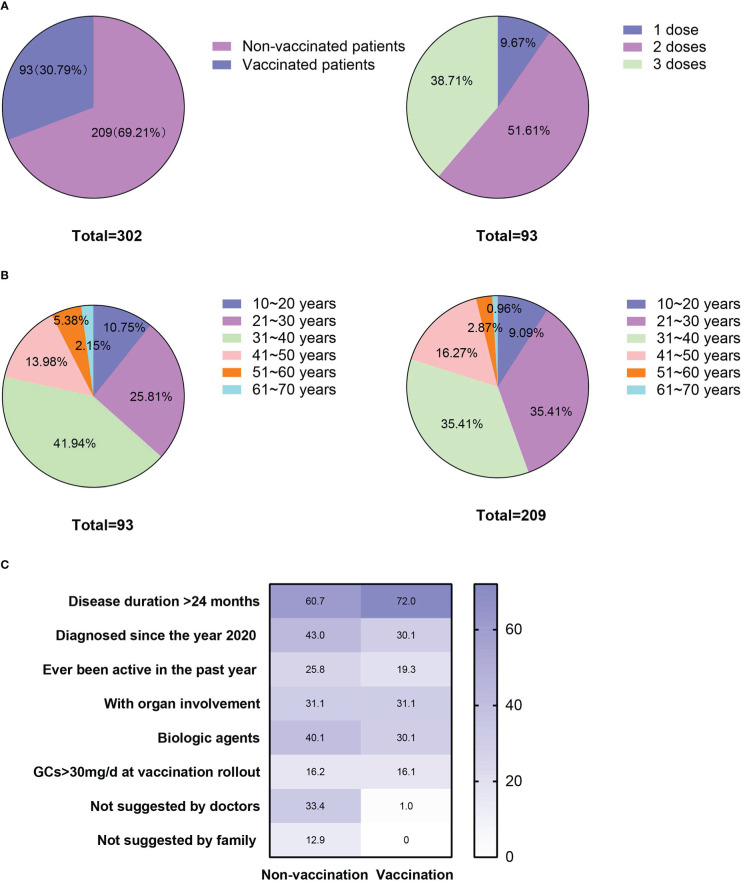
Vaccination status, age distributions and factors that might have affected patients’ vaccination status. **(A)** Patients’ vaccination status and the number of doses accepted; **(B)** Age distribution of the vaccinated and unvaccinated patients; and **(C)** Percentages of the factors that might have affected the vaccination status of the patients in the vaccinated and unvaccinated groups.

Among the 302 patients who responded to the questionnaire, 93 (30.79%) accepted the COVID-19 vaccination, nine (2.98%) received one dose, 48 (15.98%) received two doses, and 36 (11.92%) received three doses with an additional booster (**Figure 1A**). None of them had SARS‐CoV‐2 infection.

Patients’ underlying reasons for not receiving the vaccination are presented in **Table 2**. The most common reasons for vaccine hesitation among the 209 unvaccinated patients was their concern about side effects (136, 65.07%), followed by fear of disease relapse (94, 44.98%). As per their treating physicians’ recommendations against vaccination, 52 (24.88%) patients were not vaccinated and 50 (23.92%) were not vaccinated due to poorly controlled or uncontrolled diseases. Co-occurring disorders, such as cardiac disease and pregnancy or preparing for pregnancy (preconception health care) were also reasons for remaining unvaccinated (**Table 2**).

**Table 2:** COVID-19-vaccination hesitancy reasons among 209 patients with Takayasu’s arteritis.

Reasons for vaccine hesitancy	Percentage, n (%)
Concerned about side effects	136 (65.07)
Concerned about disease relapse	94 (44.98)
Not recommended by the attending doctors	52 (24.88)
Uncontrolled disease condition	50 (23.92)
Unwillingness to get the vaccination	19 (9.09)
Not recommended by family members	13 (6.22)
Concurrent other disorders, such as hypertension, uremia and coronary heart disease	10 (4.78)
Pregnancy or preparing for pregnancy	4 (1.91)
Unknown whether it is suitable to be vaccinated	2 (0.96)
Fear of vaccination	2 (0.96)
Not necessary to be vaccinated	2 (0.96)
Concurrent immunosuppressive treatment	2 (0.96)
TAK onset after flu vaccination	1 (0.48)
Taking multiple medications	1(0.48)

TAK, Takayasu’s arteritis.

### Demographic and clinical characteristics of COVID-19 vaccinated and unvaccinated patients

Characteristics of the vaccinated and unvaccinated patients are presented in **Table 1**. No difference was observed in their age (33.59 ± 14.38 vs. 33.91 ± 10.68, p = 0.84), age distribution (p = 0.45) or gender ratio (females vs males: 179, 85.65% vs. 85, 91.40%, p = 0.19) at the vaccination rollout (**Table 1**, **Figure 1B**). Compared with unvaccinated patients, the vaccinated patients had a relatively longer disease duration (p = 0.08; **Table 1**) and a lower percentage of recent diagnosis (since the year 2020, p = 0.04; **Table 1**). No differences were found in imaging type or organ involvement between the two groups. However, treatment regimens differed significantly between them (p < 0.001, **Table 1**). Specifically, the percentage of patients treated with GCs and biologic DMARDs was higher in the unvaccinated group than in the vaccinated group (84, 40.19% vs. 17, 18.28%, respectively; p < 0.001). The percentages of the multiple factors that might have affected the vaccination status of patients in the vaccinated and unvaccinated groups are shown in **Figure 1C**.

### COVID-19 vaccination uptake rate of patients with different clinical features

The vaccine-acceptance rate among patients with different clinical features are shown in **Table 3**. Among the patients with different imaging types, those with type III had the highest vaccination rate (6, 46.15%), while patients with type IIA had the lowest vaccination rate (1, 8.33%). However, no difference in the vaccination rate was found between patients with and without organ involvement (66, 31.43% vs. 27, 29.35%, p = 0.13). Among patients with different treatment regimens, the vaccine-acceptance rates were successively reduced among those treated with single GCs (5, 38.46%), conventional DMARDs (53, 31.18%) and biologic DMARDs (17, 16.83%).

**Table 3:** Vaccination rates of patients with different clinical features.

Clinical feature	Total number (N)	Number, percentage, n (%)
Imaging types, n (%)		
I	85	27 (31.76)
IIA	12	1 (8.33)
IIB	43	16 (37.21)
III	13	6 (46.15)
IV	17	7 (41.18)
V	132	36 (27.27)
Organ involvement, n (%)		
Without	210	66 (31.43)
With	92	27 (29.35)
Cerebral infarction	28	7 (25.00)
Pulmonary infarction	4	1 (25.00)
Heart failure or cardiac infarction	25	10 (40.00)
Renal dysfunction or renal atrophy	35	9 (25.71)
Major treatment		
GCs and biologic DMARDs, n (%)	101	17 (16.83)
TOF	40	10 (25.00)
TCZ	33	5 (15.15)
ADA	21	1 (4.76)
IL17Ab	7	1 (14.28)
GCs and conventional DMARDs, n (%)	170	53 (31.18)
LEF	85	18 (21.18)
MTX	20	7 (35.00)
MMF	15	7 (46.67)
AZA	12	5 (41.67)
CTX	6	3 (50.00)
HCQ	14	4 (28.57)
Others*	18	9 (50.00)
**Single GCs**	13	5 (38.46)
**No treatment**	18	18 (100.00)

ESR, erythrocyte sedimentation rate; CRP, C-reactive protein; DMARDs, disease-modifying anti-rheumatic drugs; ADA, adalimumab; TCZ, Tocilizumab; AZA, Azathioprine; TOF, tofacitinib; LEF, leflunomide; MMF, mycophenolate mofetil; IL17Ab, Secukinumab; MTX, methotrexate; CTX, cyclophosphamide; HCQ, hydroxychloroquine; GCs, glucocorticoids; *Others: curcumin, Tacrolimus and sirolimus.

### Side effects of COVID-19 vaccination and their potential effects on disease activity

Among the 93 vaccinated patients, 16 (17.20%) developed side effects, including headache or dizziness, fever, neck pain and abdominal pain (**Table 4**). The most common side effect was headache (11, 11.83%), followed by fever (5, 5.38%) and neck pain (3, 3.22%). All of these side effects were mild and did not require clinical care.

**Table 4:** COVID-19 vaccination side effects of 93 vaccinated patients.

Side effect	Percentage, n (%)
Headache or dizziness	11 (11.83)
Fever	5 (5.38)
Neck pain	3 (3.22)
Abdominal pain	2 (2.15)
Myalgia	2 (2.15)
Skin itch	2 (2.15)
Increase of ESR and CRP	2 (2.15)
Cough	1 (1.08)
Tinnitus	1 (1.08)
Discomfort of upper limb	1 (1.08)
Palpitation	1 (1.08)
Injection site swelling and redness	1 (1.08)

ESR, erythrocyte sedimentation rate; CRP, C-reactive protein.

**Figure 2:**
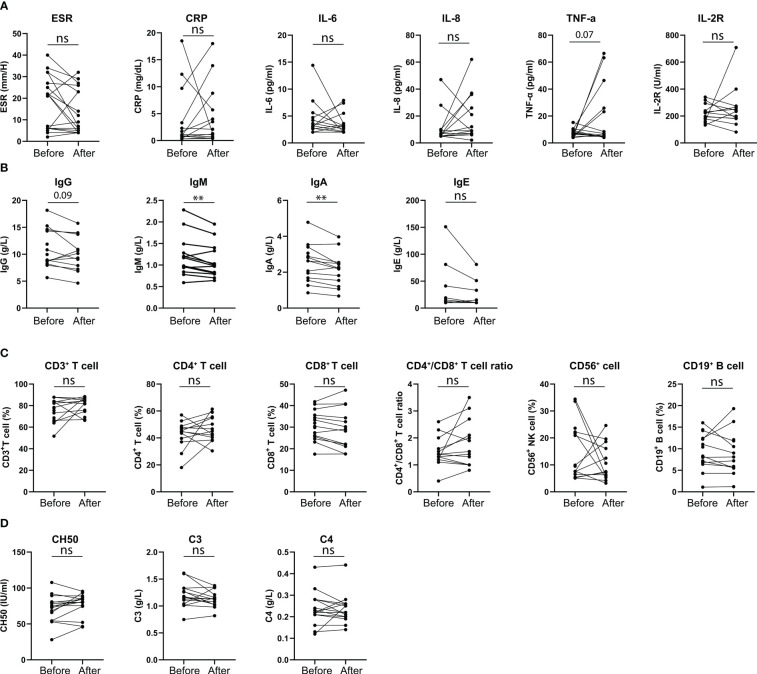
Clinical characteristics of patients with active disease post COVID-19 vaccination. **(A)** Number of active patients post different doses of vaccination. **(B)** Days of occurrence of active disease from patients’ last vaccination dose. **(C)** Patients’ symptoms at disease activation. **(D)** Patients’ CRP, ESR and IL-6 levels at disease activation.

However, eight (8.60%) patients developed TAK-associated new-onset symptoms or worsening of symptoms. Their specific information is shown in **Figure 2** and **Table 5**. Among them, 6 cases (Patient 3 to 8, **Table 5**) were newly diagnosed after vaccination and 2 cases (Patient 1 and 2, **Table 5**) were previously diagnosed and developed new symptoms after vaccination. Furthermore, among the 6 newly diagnosed TAK patients after vaccination, 5 cases (Patient 3 to 5 and patient 7 to 8, **Table 5**) developed first symptoms of TAK after vaccination and 1 case (Patient 6, **Table 5**) suffered from worsened previous symptom after vaccination and then was diagnosed. Side effects occurred in six patients within one month (median 17.5 [14.25, 22.5] days) after the last vaccine dose (Patients 1-6) and developed approximately three months later in the remaining two patients (Patients 7-8). Two (2.15%) patients developed serious adverse effects (vision defect in Patient 2 and aphasia in Patient 7). Moderate and mild increases in the ESR or CRP were observed in the eight patients, and all of them were assessed as active status after hospitalization. One of the two previously diagnosed patients changed the individualized treatment regimen to GCs combined with MTX and Secukinumab (Patient 2). The six newly diagnosed patients initiated immunosuppressive therapy. Two of them were treated with GCs combined with MTX and biologic agents due to the severity of their conditions.

**Table 5:** Clinical characteristics of the patients with new-onset or worsening of their symptoms after the vaccination.

Characteristic	Patient 1	Patient 2	Patient 3	Patient 4	Patient 5	Patient 6	Patient 7	Patient 8
Age (years)	33	38	20	33	14	40	27	38
Gender (Female, male)	F	F	F	F	F	F	F	F
Disease duration	14 years	4 years	1 months	8 months	/	1 year	/	/
TAK diagnosis before or after vaccination	Before	Before	After	After	After	After	After	After
NIH score	2	2	3	2	2	2	2	3
New-onset or worsening of symptoms	New-onset	New-onset	New-onset	New-onset	New-onset	Worsened	New-onset	New-onset
Imaging type	IV	IIB	I	V	IIB	V	V	V
Treatment	GCs 10 mg qd +LEF 10 mg bid+Tofacitinib	GCs 40 mg qd+MTX 15 mg qweek+Secukinumab 150 mg qweek	GCs 40 mg qd +MTX 15 mg qweek	GCs 20 mg qd and AZA 25 mg qd	GCs 40 mg qd+ MTX 15 qweek	GCs 30 mg qd+MTX 15 mg qweek+Secukinumab 150 mg qweek	GCs 35 mg qd+ MTX 15 mg qweek+ADA 40 mg q2w	GCs 40 mg qd+ MTX 15 qweek
Follow-up	Improved	Improved	Improved	Improved	Improved	Improved	Improved	Improved

TAK, Takayasu’s arteritis; NIH, National Institutes of Health; qd, daily; bid, twice a day; qweek, every week; q2weeks, every 2 weeks.

Among 209 patients without vaccination, 54 (25.84%) patients experienced relapses during 1^st^ May, 2021 to 30 April, 2022.

### Changes of parameters related with patients’ immune functions after vaccination

**Figure 3:**
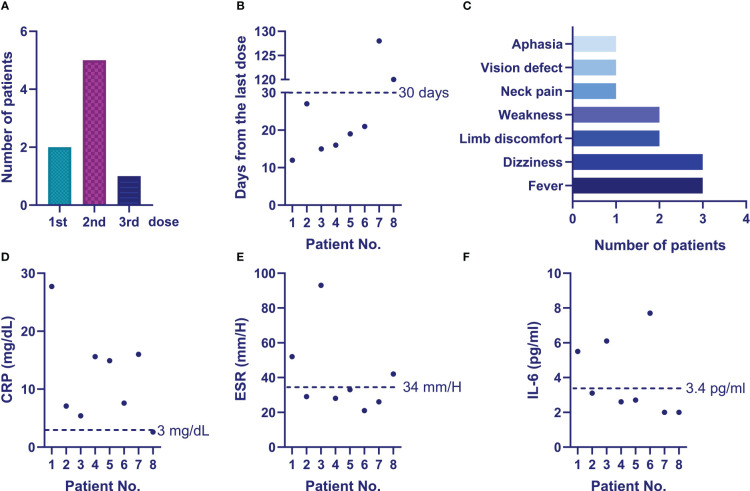
Changes in the immune-related parameters of the vaccinated patients. **(A)** Changes of patients’ inflammatory parameters before and after vaccination; **(B)** Changes of patients’ immunoglobulins before and after vaccination; **(C)** Changes of patients’ peripheral immune cell percentages before and after vaccination; **(D)** Changes of patients’ complement before and after vaccination.

Changes in cytokines, immunoglobulin and immune cell percentages before and after vaccination were analyzed in 17 patients (**Figure 3**), who had taken at least two doses of the vaccination. Their clinical features and treatment information are presented in **Supplemental Table S2**. Their lab results were collected within a median duration of 5 (2, 6) months before the first dose and 2 (1, 3) months after the second dose. The results indicated significant decreases in their IgM (p = 0.009) and IgA (p = 0.005) levels after vaccination (**Figure 3B**). A downward trend in the IgG level was observed (p = 0.09, **Figure 3B**), but no changes were found in the other parameters, including cytokines, immune cell percentages and complement (**Figures 3A, C, D**). No significant relationship was observed between the decreased IgM or IgA and a specific treatment.

### Potential effects of COVID-19 vaccination on patients’ disease onset

Among the study’s 302 patients, 18 (5.96%) were diagnosed post-vaccination. The median duration from the last dose of the vaccination to the diagnosis was 97 (26, 118) days. These patients included the 6 cases with new or worsening symptoms after vaccination mentioned above (Patient 3-8 in **Table 5**). The other 12 newly diagnosed patients with previous symptoms did not complain of worsened or new symptoms after vaccination, but just had confirmed diagnosis after vaccination. In comparison with the 21 patients diagnosed during the same period but without prior vaccination, these patients had higher percentages of the CD19^+^ B cell subset (p = 0.04, **Supplemental Table S3**), and patients with a prior COVID-19 vaccination tended to have lower IL-6 (p = 0.09), TNF-α (p = 0.07) and IL-8 levels (p = 0.08). No significant differences between patients with and without a prior vaccination were found in the other clinical features, including patients’ activity, imaging types, immunoglobulin or complement levels (**Supplemental Table S3**).

## Discussion

This study analyzed COVID-19 vaccination rates, vaccine hesitancy and the potential clinical effects of vaccination in a cohort of 302 patients with TAK. The results indicate that the vaccination rate in patients with TAK was relatively low (30.79%). Most of the patients were reluctant to accept the vaccination due to their fear of side effects or disease flares. Approximately 17.2% of the vaccinated patients developed side effects. Eight (8.6%) patients presented with worsening of symptoms or new-onset symptoms post-vaccination, which were assessed as disease flares or a new active disease.

According to previous reports, the COVID-19 vaccination rate of patients with rheumatic disease ranged from 24.1% to 78.9% across different countries (11, 12). The vaccination rate in this study (30.79%) was comparable to the lower rates previously reported. In this study, the most common reason for not being vaccinated were concerns about side effects and disease flares. These reasons were also common in other investigations of patients with rheumatic diseases (12, 13). In this study, we also analyzed potential clinical characteristics that might have affected patients’ vaccination status. After comparing vaccinated and unvaccinated patients, the results showed that patients with a shorter duration of disease and those receiving biologic treatments were less likely to be vaccinated. These patients might have been more concerned about the impact of the vaccine’s side effects on their disease or they might have behaved with more caution due to their biologic treatment. However, other features, such as age, disease activity, severe disease with organ involvement and imaging types were irrelevant to the vaccination status of the patients.

Since the breakout of the COVID-19 epidemic, numerous questions and consults from patients have been received about whether they should be vaccinated. To answer these questions, this study analyzed the side effects of the COVID-19 vaccination and its potential effects on their diseases. The vaccines’ incidence of side effects was low (17.20%) and most of the symptoms were mild. The most common systemic side effects were headache (11.83%), followed by fever (5.38%). However, eight (8.60%) patients experienced disease flares or disease onset due to worsening of their symptoms or the development of new symptoms. Two (2.15%) patients had serious adverse effects, one with a vision defect and one with cranial infarction. According to previous reports, vaccination-related thrombosis is not considered a rare side effect (14). As TAK is an inflammatory vascular disease, it would be worthwhile to investigate whether a patient with TAK is more likely to develop thrombosis. Fortunately, the symptoms of the patients in the present study improved during follow-up after treatment.

To understand the side effects of COVID-19 vaccination in other rheumatic diseases, twenty-four studies have been reviewed. The literature review strategy is shown in **Supplementary material** and the corresponding information are summarized in **Supplemental Table S4**. Four studies included information about inactivated vaccinations (15–18) and the remaining studies included mRNA or viral vector vaccinations. The total incidence of side effects in patients with rheumatic disease was approximately 29.9%-89.0%. Most of the side effects were localized reactions (8.1%-89.0%), which predominantly presented as pain. The incidence of systemic side effects was as high as 80.1%, including fatigue, myalgia, headache and fever. Among them, fatigue was the most common (9.4%-80.0%) side effect, followed by headache (2.0%-52.1%) and myalgia (9.4%-29.3%). Approximately 5.0%-23.27% of patients presented with fever after a COVID-19 vaccination.

Only five studies reported severe adverse effects (0.4-5.7%) of the vaccination, which included thromboembolic events, uveitis, myocarditis and pericarditis (17, 19–22); mortality was not observed. Thirteen (0.23%-16.0%) studies reported disease flares (12, 16–20, 23–29), and five of them analyzed possible risk factors (16, 24, 26, 28, 29). Only one study indicated that the ChAdOx1 nCoV-19 vaccination may have posed a higher risk for disease flares than the BNT162b2 mRNA vaccination (28). The other factors examined in this study were all related to disease conditions or patients’ demographic characteristics. Other studies all concluded that the vaccination was safe (30–34) and didn’t increase the risk for disease flare (35). Only two studies included patients with TAK, but they did not provide a detailed description of their side effects or flares (19, 36). Hence, the incidence of side effects and disease flares in patients with TAK in this study were all within the range of those reported in most of the studies on rheumatic diseases. Thus, there was not any indication not to vaccine against COVID-19 for patients with TAK and that doctors should be advised not to discourage their patients with TAK.

Vaccinations take effect by stimulating the body to produce antibodies; thus, it might affect immunoglobulin levels and other immune functions. By analyzing multiple immune-related parameters before and after the vaccinations of 17 patients, we observed decreases in IgA, IgM and a downward trend in IgG. As shown in **Supplementary Table S2**, all the 17 patients were already on immunosuppressive treatments including methotrexate, tocilizumab, leflunomide, mycophenolate mofetil before vaccination. According to current research (37), these agents could weaken the immunogenicity of the vaccination. In addition, at the second estimation, 94.12% patients were in stable status. All these factors might affect the production of immunoglobulins at the second estimation (after vaccination). Furthermore, since specific IgG antibodies against SARS-CoV-2 were not detected in the present study, further investigation is needed to determine whether decreases in these immunoglobulins are related to COVID-19 vaccinations.

Whether COVID-19 vaccination might affect patients’ signs and symptoms and inflammatory or immune parameters at disease onset was also analyzed. By comparing clinical characteristics at disease onset between patients with and without prior COVID-19 vaccination during the same period, we found that patients with prior vaccinations had a higher CD19^+^ B cell percentage, which indicates a positive effect of the vaccination on patients’ B cells.

In addition, relatively lower levels of cytokines, such as IL-6, TNF-α and IL-8 were found in patients with vaccination in contrast to the patients without prior vaccination. The cytokines were all detected before their treatments. As shown in **Supplementary Table S3**, the unvaccinated patients complained of more ischemic or systemic symptoms and active disease status compared with vaccinated patients, which might indicate that unvaccinated patients had more severe disease status at the onset. Thus, the cytokines detected in vaccinated patients were lower compared to those unvaccinated, this was also consistent with their ESR or CRP levels.

At last, the response rate was 64.95% in this study. According to recently publised studies about COVID-19 vaccination survey in Chinese population, the response rate varied largely from 23% to 77.4% (38–41). Thus, the response rate might differ due to disease population, cohort types, etc. Our study has a fairly acceptable response rate.

The limitations of this study included several aspects. First, this survey was retrospectively conducted, and the validations and reliability of the questionnaire haven’t been determined; thus, the results of this study needs to be validated in future research. Second, whether COVID-19 vaccinations had a direct effect on patients’ disease flares require additional verification. Last, as the number of patients with vaccinations was relatively small, including the patients with follow up immune parameters, research on a larger number of patients is warranted to confirm the results of this study.

In conclusion, the vaccination rate was low in TAK, which was mainly caused by concerns about negative effects of vaccination on their disease. An acceptable safety profile was observed in vaccinated patients. The risk of disease flare associated with COVID-19 vaccination warrants further investigation.

## Data availability statement

The original contributions presented in the study are included in the article/**Supplementary Material**. Further inquiries can be directed to the corresponding author.

## Ethics statement

The studies involving human participants were reviewed and approved by Institutional Review Board of Zhongshan Hospital, Fudan University. The patients/participants provided their written informed consent to participate in this study.

## Author contributions

XK was responsible for the data analysis and manuscript writing. XD was responsible for the distribution of the questionnaire. LM collected the data of the questionnaire. JW and YS collected patients’ clinical data. LJ was responsible for the whole study design. All authors contributed to the article and approved the submitted version.

## References

[1] Li M Wang H Tian L Pang Z Yang Q Huang T. COVID-19 vaccine development: milestones, lessons and prospects. Signal Transduct Target Ther (2022) 7:146. doi: 10.1038/s41392-022-00996-y 35504917 PMC9062866

[2] Tregoning JS Flight KE Higham SL Wang Z Pierce BF. Progress of the COVID-19 vaccine effort: viruses, vaccines and variants versus efficacy, effectiveness and escape. Nat Rev Immunol (2021) 21(10):626–36. doi: 10.1038/s41577-021-00592-1 PMC8351583 34373623

[3] Mathieu E Ritchie H Rodés-Guirao L Appel C Giattino C Hasell J. Coronavirus pandemic (COVID-19). OurWorldInData.org (2020).

[4] Cho K Park S Kim EY Koyanagi A Jacob L Yon DK. Immunogenicity of COVID-19 vaccines in patients with diverse health conditions: A comprehensive systematic review. J Med Virol (2022) 94(9):4144–55. doi: 10.1002/jmv.27828 PMC9347877 35567325

[5] Esatoglu SN Hatemi G. Takayasu arteritis. Curr Opin Rheumatol (2022) 34(1):18–24. doi: 10.1097/BOR.0000000000000852 34698679

[6] Maz M Chung SA Abril A Langford CA Gorelik M Guyatt G. 2021 American College of rheumatology/ vasculitis foundation guideline for the management of giant cell arteritis and takayasu arteritis. Arthritis Rheumatol (2021) 73(8):1349–65. doi: 10.1002/art.41774 PMC12344528 34235884

[7] Hyrich KL Machado PM. Rheumatic disease and COVID-19: epidemiology and outcomes. Nat Rev Rheumatol (2021) 17(2):71–2. doi: 10.1038/s41584-020-00562-2 PMC7747184 33339986

[8] Arend WP Michel BA Bloch DA Hunder GG Calabrese LH Edworthy SM. The American college of rheumatology 1990 criteria for the classification of takayasu arteritis. Arthritis Rheum (1990) 33(8):1129–34. doi: 10.1002/art.1780330811 1975175

[9] Kerr GS Hallahan CW Giordano J Leavitt RY Fauci AS Rottem M. Takayasu arteritis. Ann Intern Med (1994) 120(11):919–29. doi: 10.7326/0003-4819-120-11-199406010-00004 7909656

[10] Hata A Noda M Moriwaki R Numano F. Angiographic findings of takayasu arteritis: new classification. Int J Cardiol (1996) 54(Suppl):S155–63. doi: 10.1016/s0167-5273(96)02813-6 9119518

[11] Li X Tong X Yeung WWY Kuan P Yum SHH Chui CSL. Two-dose COVID-19 vaccination and possible arthritis flare among patients with rheumatoid arthritis in Hong Kong. Ann Rheum Dis (2022) 81(4):564–68. doi: 10.1136/annrheumdis-2021-221571 PMC8550868 34686479

[12] Fragoulis GE Bournia VK Mavrea E Evangelatos G Fragiadaki K Karamanakos A. COVID-19 vaccine safety and nocebo-prone associated hesitancy in patients with systemic rheumatic diseases: a cross-sectional study. Rheumatol Int (2022) 42(1):31–9. doi: 10.1007/s00296-021-05039-3 PMC8569844 34739573

[13] Gaur P Agrawat H Shukla A. COVID-19 vaccine hesitancy in patients with systemic autoimmune rheumatic disease: an interview-based survey. Rheumatol Int (2021) 41(9):1601–05. doi: 10.1007/s00296-021-04938-9 PMC8249840 34213580

[14] Pottegard A Lund LC Karlstad Ø Dahl J Andersen M Hallas J. Arterial events, venous thromboembolism, thrombocytopenia, and bleeding after vaccination with Oxford-AstraZeneca ChAdOx1-s in Denmark and Norway: population based cohort study. BMJ (2021) 373:n1114. doi: 10.1136/bmj.n1114 33952445 PMC8097496

[15] Medeiros-Ribeiro AC Aikawa NE Saad CGS Yuki EFN Pedrosa T. Immunogenicity and safety of the CoronaVac inactivated vaccine in patients with autoimmune rheumatic diseases: a phase 4 trial. Nat Med (2021) 27(10):1744–51. doi: 10.1038/s41591-021-01469-5 34331051

[16] Fan Y Geng Y Wang Y Deng X Li G Zhao J. Safety and disease flare of autoimmune inflammatory rheumatic diseases: a large real-world survey on inactivated COVID-19 vaccines. Ann Rheum Dis (2022) 81(3):443–45. doi: 10.1136/annrheumdis-2021-221736 PMC8862022 34824048

[17] Ozdede A Guner S Ozcifci G Yurttas B Toker Dincer Z Atli Z. Safety of SARS-CoV-2 vaccination in patients with behcet's syndrome and familial Mediterranean fever: a cross-sectional comparative study on the effects of m-RNA based and inactivated vaccine. Rheumatol Int (2022) 42(6):973–87. doi: 10.1007/s00296-022-05119-y PMC8977433 35376962

[18] Cherian S Paul A Ahmed S Alias B Manoj M Santhosh AK. Safety of the ChAdOx1 nCoV-19 and the BBV152 vaccines in 724 patients with rheumatic diseases: a post-vaccination cross-sectional survey. Rheumatol Int (2021) 41(8):1441–45. doi: 10.1007/s00296-021-04917-0 PMC8211311 34142203

[19] Machado PM Lawson-Tovey S Strangfeld A Mateus EF Hyrich KL Gossec L. Safety of vaccination against SARS-CoV-2 in people with rheumatic and musculoskeletal diseases: results from the EULAR coronavirus vaccine (COVAX) physician-reported registry. Ann Rheum Dis (2022) 81(5):695–709. doi: 10.1136/annrheumdis-2021-221490 34972811

[20] Lee TJ Lu CH Hsieh SC. Herpes zoster reactivation after mRNA-1273 vaccination in patients with rheumatic diseases. Ann Rheum Dis (2022) 81(4):595–97. doi: 10.1136/annrheumdis-2021-221688 34876461

[21] Bartels LE Ammitzboll C Andersen JB Vils SR Mistegaard CE Johannsen AD. Local and systemic reactogenicity of COVID-19 vaccine BNT162b2 in patients with systemic lupus erythematosus and rheumatoid arthritis. Rheumatol Int (2021) 41(11):1925–31. doi: 10.1007/s00296-021-04972-7 PMC8412379 34476603

[22] Furer V Eviatar T Zisman D Peleg H Paran D Levartovsky D. Immunogenicity and safety of the BNT162b2 mRNA COVID-19 vaccine in adult patients with autoimmune inflammatory rheumatic diseases and in the general population: a multicentre study. Ann Rheum Dis (2021) 80(10):1330– 38. doi: 10.1136/annrheumdis-2021-220647 34127481

[23] Barbhaiya M Levine JM Bykerk VP Jannat-Khah D Mandl LA. Systemic rheumatic disease flares after SARS-CoV-2 vaccination among rheumatology outpatients in new York city. Ann Rheum Dis (2021) 80(10):1352–54. doi: 10.1136/annrheumdis-2021-220732 34158370

[24] Connolly CM Ruddy JA Boyarsky BJ Barbur I Werbel WA Geetha D. Disease flare and reactogenicity in patients with rheumatic and musculoskeletal diseases following two-dose SARS-CoV-2 messenger RNA vaccination. Arthritis Rheumatol (2022) 74(1):28–32. doi: 10.1002/art.41924 34346185 PMC8712346

[25] Syversen SW Jyssum I Tveter AT Tran TT Sexton J Provan SA. Immunogenicity and safety of standard and third-dose SARS-CoV-2 vaccination in patients receiving immunosuppressive therapy. Arthritis Rheumatol (2022) 74(8):1321–32. doi: 10.1002/art.42153 PMC9347774 35507355

[26] Rotondo C Cantatore FP Fornaro M Colia R Busto G Rella V. Preliminary data on post market safety profiles of COVID 19 vaccines in rheumatic diseases: Assessments on various vaccines in use, different rheumatic disease subtypes, and immunosuppressive therapies: A two-centers study. Vaccines (Basel) (2021) 9(7):1–11. doi: 10.3390/vaccines9070730 PMC8310114 34358147

[27] Sattui SE Liew JW Kennedy K Sirotich E Putman M Moni TT. Early experience of COVID-19 vaccination in adults with systemic rheumatic diseases: results from the COVID-19 global rheumatology alliance vaccine survey. RMD Open (2021) 7(3):1–10. doi: 10.1136/rmdopen-2021-001814 PMC8424419 34493645

[28] Rider LG Parks CG Wilkerson J Schiffenbauer AI Kwok RK Noroozi Farhadi P. Baseline factors associated with self-reported disease flares following COVID-19 vaccination among adults with systemic rheumatic disease: results from the COVID-19 global rheumatology alliance vaccine survey. Rheumatol (Oxford) (2022) 61(SI2):I143–50. doi: 10.1093/rheumatology/keac249 PMC9248066 35460240

[29] Spinelli FR Favalli EG Garufi C Cornalba M Colafrancesco S Conti F. Low frequency of disease flare in patients with rheumatic musculoskeletal diseases who received SARS-CoV-2 mRNA vaccine. Arthritis Res Ther (2022) 24(1):21. doi: 10.1186/s13075-021-02674-w 35016701 PMC8748531

[30] Esquivel-Valerio JA Skinner-Taylor CM Moreno-Arquieta IA Cardenas-de la Garza JA Garcia-Arellano G Gonzalez-Garcia PL. Adverse events of six COVID-19 vaccines in patients with autoimmune rheumatic diseases: a cross-sectional study. Rheumatol Int (2021) 41(12):2105–08. doi: 10.1007/s00296-021-05017-9 PMC8496432 34622311

[31] Li YK Lui M Yam LL Cheng CS Tsang THT Kwok WS. COVID-19 vaccination in patients with rheumatic diseases: Vaccination rates, patient perspectives, and side effects. Immun Inflammation Dis (2022) 10(3):e589. doi: 10.1002/iid3.589 PMC8926511 35099852

[32] Tzioufas AG Bakasis AD Goules AV Bitzogli K Cinoku II Chatzis LG. A prospective multicenter study assessing humoral immunogenicity and safety of the mRNA SARS-CoV-2 vaccines in Greek patients with systemic autoimmune and autoinflammatory rheumatic diseases. J Autoimmun (2021) 125:102743. doi: 10.1016/j.jaut.2021.102743 34757289 PMC8552665

[33] Boekel L Kummer LY van Dam KPJ Hooijberg F van Kempen Z Vogelzang EH. Adverse events after first COVID-19 vaccination in patients with autoimmune diseases. Lancet Rheumatol (2021) 3(8):e542–45. doi: 10.1016/S2665-9913(21)00181-8 PMC8213359 34179831

[34] Connolly CM Ruddy JA Boyarsky BJ Avery RK Werbel WA Segev DL. Safety of the first dose of mRNA SARS-CoV-2vaccines inpatients with rheumatic and musculoskeletal diseases. Ann Rheum Dis (2021) 80(8):1100–01. doi: 10.1136/annrheumdis-2021-220231 PMC10105326 33741555

[35] Pinte L Negoi F Ionescu GD Caraiola S Balaban DV Badea C. COVID-19 vaccine does not increase the risk of disease flare-ups among patients with autoimmune and immune-mediated diseases. J Pers Med (2021) 11(12):1283. doi: 10.3390/jpm11121283 34945754 PMC8707188

[36] Braun-Moscovici Y Kaplan M Braun M Markovits D Giryes S Toledano K. Disease activity and humoral response in patients with inflammatory rheumatic diseases after two doses of the pfizer mRNA vaccine against SARS-CoV-2. Ann Rheum Dis (2021) 80(10):1317–21. doi: 10.1136/annrheumdis-2021-220503 34144967

[37] Curtis JR Johnson SR Anthony DD Arasaratnam RJ Baden LR Bass AR. American College of rheumatology guidance for COVID-19 vaccination in patients with rheumatic and musculoskeletal diseases: Version 4. Arthritis Rheumatol (2022) 74(5):e21–36. doi: 10.1002/art.42109 PMC9082483 35474640

[38] Zhou Y Lin Z Wan X Liu J Ding J Zhang C. COVID-19 vaccine acceptance and hesitancy in patients with parkinson's disease. Front Public Health (2022) 10:977940. doi: 10.3389/fpubh.2022.977940 36304248 PMC9595444

[39] Wong MCS Wong ELY Huang J Cheung AWL Law K Chong MKC. Acceptance of the COVID-19 vaccine based on the health belief model: A population-based survey in Hong Kong. Vaccine (2021) 39(7):1148–56. doi: 10.1016/j.vaccine.2020.12.083 PMC7832076 33461834

[40] Lin XQ Zhang MX Chen Y Xue JJ Chen HD Tung TH. Relationship between knowledge, attitudes, and practices and COVID-19 vaccine hesitancy: A cross-sectional study in taizhou, China. Front Med (Lausanne) (2022) 9:770933. doi: 10.3389/fmed.2022.770933 36082277 PMC9445127

[41] Zhang KC Fang Y Cao H Chen H Hu T Chen Y. Behavioral intention to receive a COVID-19 vaccination among Chinese factory workers: Cross-sectional online survey. J Med Internet Res (2021) 23(3):e24673. doi: 10.2196/24673 33646966 PMC7945977

