# Supplementary material for: COVID-19 vaccine uptake, hesitancy and clinical effects on patients with Takayasu’s arteritis: A web-based questionnaire survey from a large cohort
Source: Front Immunol. 2023 Feb 9;14:1030810. doi: 10.3389/fimmu.2023.1030810 (PMC9946967; doi:10.3389/fimmu.2023.1030810)
Supplement: Supplementary file 1 [file DataSheet_1.docx]

**Supplementary Figure S1. A flow-chart of this study**


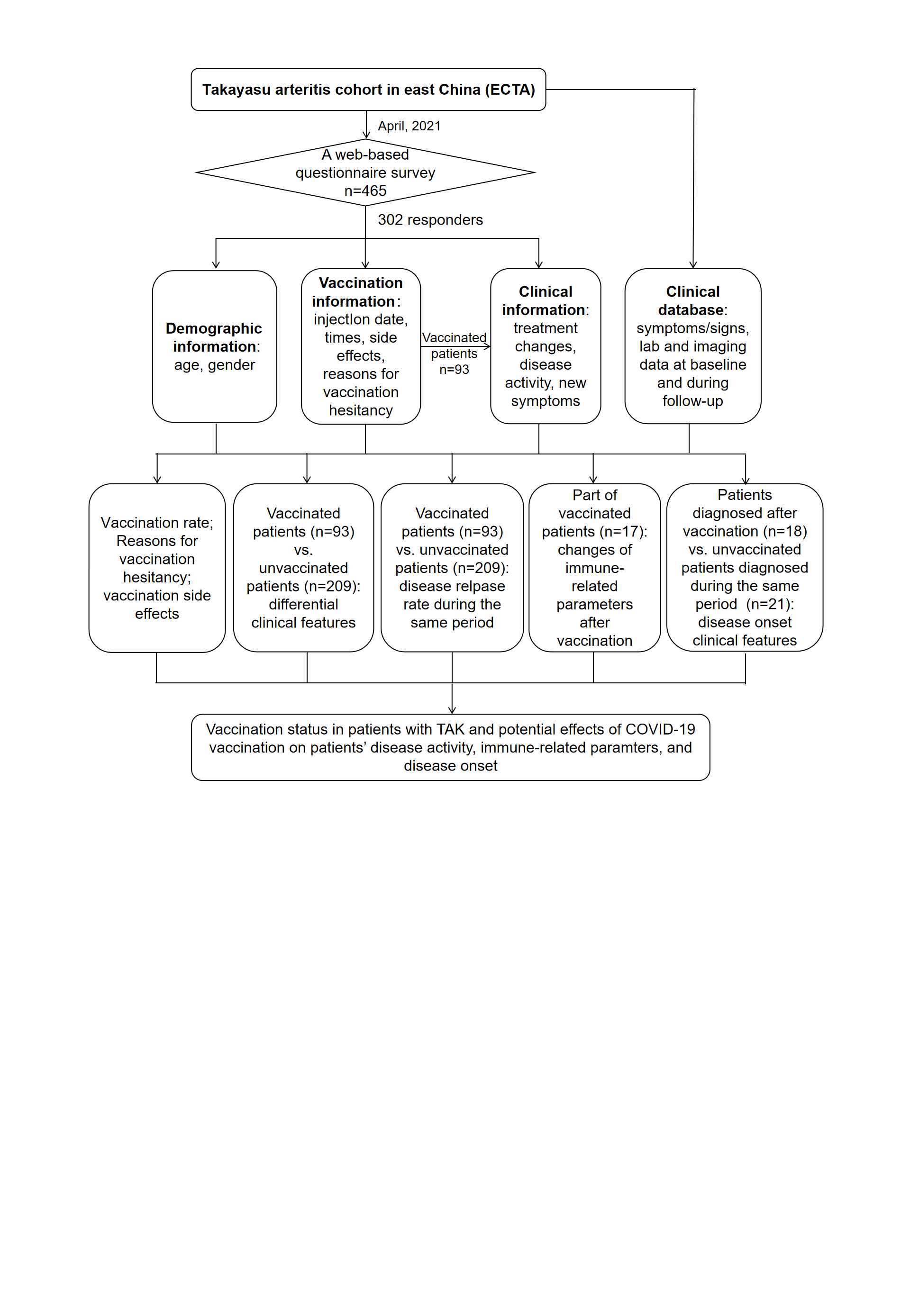


**Supplemental Table S1. Characteristics of the responders and non-responders to the vaccination survey**

| **Characteristics** | **Responders**  **(n=302)** | **Non-responders**  **（n=163）** | **p** |
| --- | --- | --- | --- |
| **Age at diagnosis (mean ± SD, years)** | 30.59±10.30 | 32.43±12.17 | 0.11 |
| **Age at vaccination rollout (mean ± SD, years)** | 33.69±13.32 | 34.26±10.04 | 0.50 |
| **Sex (female: male, ratio)** | 264: 38 | 133:30 | 0.10 |
| **Disease duration (median, IQR, months)** | 35 (16.25, 58.00) | 40.17 (14.40, 78.14) | 0.23 |
| **Been active in the past year, n (%)** | 72 (23.84) |  |  |
| **Imaging types, n (%)** |  |  | 0.72 |
| I | 85 (28.14) | 48 (29.45) |  |
| IIA | 12 (3.97) | 8 (4.91) |  |
| IIB | 43 (14.24) | 21 (12.88) |  |
| III | 13 (4.30) | 7 (4.29) |  |
| IV | 17 (5.63) | 15 (9.20) |  |
| V | 132 (43.71) | 64 (39.26) |  |
| **TAK-associated organ involvement, n (%)** | 92 (30.46) | 36 (22.09) | 0.06 |
| Cerebral infarction | 28 (9.27) | 7 (4.29) |  |
| Pulmonary infarction | 4 (1.32) | 4 (2.45) |  |
| Heart failure or cardiac infarction | 25 (8.28) | 15 (9.20) |  |
| Renal dysfunction or renal atrophy | 35 (11.59) | 10 (6.13) |  |
| **Major treatment** |  |  | 0.83 |
| GCs and Biologic DMARDs, n (%) | 101 (33.44) | 59 (36.20) |  |
| GCs and Conventional DMARDs, n (%) | 170 (56.29) | 90 (55.21) |  |
| **Single GCs** | 13 (4.30) | 6 (3.68) |  |
| **No treatment** | 18 (5.96) | 7 (4.29) |  |

TAK, takayasu arteritis; IQR, interquartile range; DMARDs, disease-modifying anti-rheumatic drugs; GCs, glucocordicoids

**Supplemental Table S2. Clinical characteristics of 17 patients before and after receiving the COVID-19 vaccination**

| **Characteristics** | **Before vaccination** | **After vaccination** |
| --- | --- | --- |
| **Age (mean±SD, years)** | 31.53±12.72 | **/** |
| **Gender (females: males, ratio)** | 15 : 2 | **/** |
| **Disease duration (median, IQR, months)** | 41.00 (29.00, 43.50) | **/** |
| **Disease activity (n, %)** | 5 (29.41) | 1 (5.88) |
| **Treatment (n, %)** |  |  |
| GCs+MTX+TCZ | 3 (17.65) | 3 (17.65) |
| GCs+TOF | 2 (11.76) | 2 (11.76) |
| GCs+LEF | 5 (29.41) | 5 (29.41) |
| GCs+MMF | 1 (5.88) | 1 (5.88) |
| GCs+MTX | 3 (17.65) | 3 (17.65) |
| GCs+HCQ | 2 (11.76) | 2 (11.76) |
| GCs+Tacrolimus | 1 (5.88) | 1 (5.88) |

GCs, glucocorticoids; MTX, methotrexate; TCZ, Tocilizumab; TOF, tofacitinib; LEF, leflunomide,

MMF: mycophenolate mofetil, HCQ: hydroxychloroquine

**Supplemental Table S3. Characteristics of the patients diagnosed in May 2021 to April 2022 with and without a prior vaccination**

| **Characteristics** | **Without a prior**  **vaccination（n=21）** | **With a prior**  **vaccination（n=18）** | **p** |
| --- | --- | --- | --- |
| **Age at diagnosis (mean ± SD, years)** | 37.95±10.05 | 33.5±12.59 | 0.53 |
| **Age at vaccination rollout (mean ± SD, years)** | 37.67±9.53 | 33.00±12.54 | 0.84 |
| **Sex (female: male, ratio)** | 18:3 | 18:0 | 0.24 |
| **Disease duration (median, IQR, months)** | 10.00 (8, 15.00) | 7.5 (5, 10.75) | 0.15 |
| **Duration between the diagnosis and the last vaccine dose (days)** | / | 97 (26, 118) | / |
| **Active disease, n (%)** | 16 (76.19) | 10 (55.56) | 0.20 |
| **Signs and symptoms, n (%)** |  |  |  |
| Ischemic symptoms | 10 (47.62) | 4 (22.22) | 0.10 |
| Systemic symptoms | 8 (38.10) | 2 (11.11) | 1.00 |
| **Lab results** |  |  |  |
| ESR (mm/H)  CRP (mg/L) | 18.00 (6.00, 39.00)  6.00 (1.05, 19.95) | 16.5 (2.00, 21.00)  1.30 (0.61, 7.60) | 0.29  0.13 |
| IL-6 (pg/ml) | 6.70 (3.50, 8.60) | 3.45 (2.88, 5.60) | **0.09** |
| TNF-α (pg/ml) | 12.2 (8.40, 31.00) | 6.15 (4.18, 14.90) | **0.07** |
| IL-8 (pg/ml) | 21.00 (11.50, 23.00) | 8.00 (6.50, 17.50) | **0.08** |
| IL-1β(pg/ml) | 6.56±2.45 | 9.40±4.75 | 0.14 |
| IL-2R (U/ml) | 333.50 (243.00, 505.25) | 241.00 (166.00, 379.00) | 0.27 |
| IgG (g/L) | 11.43±2.87 | 12.48±3.98 | 0.40 |
| IgM (g/L) | 1.46±0.59 | 1.48±0.49 | 0.90 |
| IgA (g/L)  IgE (g/L) | 2.13±0.92  35.00 (15.25, 43.75) | 2.90±1.68  21.00 (15.00, 67.00) | 0.11  0.83 |
| C3 (g/L) | 1.06±0.23 | 1.04±0.21 | 0.84 |
| C4 (g/L) | 0.22±0.07 | 0.22±0.08 | 0.80 |
| CH50 (IU/ml) | 70.32±19.78 | 65.91±15.94 | 0.55 |
| CD19 (%) | 14.88±8.69 | 21.39±7.50 | **0.04** |
| CD3 (%) | 73.64±8.86 | 68.33±10.03 | 0.14 |
| CD4 (%) | 45.98±10.05 | 40.54±11.84 | 0.19 |
| CD8 (%) | 25.27±7.15 | 24.93±7.20 | 0.89 |
| CD4/CD8 | 2.07±1.06 | 1.79±0.83 | 0.45 |
| NK (%) | 10.08±8.33 | 9.68±5.74 | 0.69 |
| **Imaging types, n(%)** |  |  |  |
| I | 5 (23.81) | 2 (11.11) | 0.35 |
| IIA | 1 (4.76) | 0 (0.00) |  |
| IIB | 2 (9.52) | 5 (27.78) |  |
| III | 0 (0.00) | 1 (5.56) |  |
| IV | 1 (4.76) | 0 (0.00) |  |
| V | 12 (57.14) | 10 (55.56) |  |
| **TAK-associated organ involvement, n (%)** | 7 (33.33) | 5 (27.78) | 0.74 |
| **Major treatment, n (%)** |  |  |  |
| GCs and biologic DMARDs | 14 (66.66) | 11 (61.11) | 0.72 |
| TOF | 1 (4.76) | 2 (11.11) |  |
| TCZ | 5 (23.81) | 3 (16.67) |  |
| ADA | 5 (23.81) | 2 (11.11) |  |
| IL17Ab | 3 (14.29) | 4 (22.22) |  |
| GCs and conventional DMARDs | 14 (66.67) | 13 (72.22) | 0.71 |
| LEF | 4 (19.05) | 1 (5.56) |  |
| MTX | 0 (0.00) | 2 (11.11) |  |
| MMF | 8 (38.10) | 7 (38.89) |  |
| AZA | 0 (0.00) | 1 (5.56) |  |
| CTX | 1 (4.76) | 0 (0.00) |  |
| HCQ | 1 (4.76) | 0 (0.00) |  |
| Others* | 0 (0.00) | 2 (11.11) |  |
| Single GCs | 1 (4.76) | 0 (0.00) | / |

ESR, erythrocyte sedimentation rate; CRP, C-reactive protein; DMARDs, disease-modifying anti-rheumatic drugs; ADA, adalimumab; TCZ, Tocilizumab; AZA, Azathioprine; TOF, tofacitinib; LEF, leflunomide; MMF, mycophenolate mofetil; IL17Ab, Secukinumab; MTX, methotrexate; CTX, cyclophosphamide; HCQ, hydroxychloroquine; GCs, glucocorticoids; *Others: curcumin, Tacrolimus or sirolimus

**Brief review of the relevant literature**

We reviewed published research on the side effects of COVID-19 vaccinations on patients with rheumatic diseases. This process was mainly based on a search of the MEDLINE database. The search terms included [“COVID-19 vaccine” or “SARS-CoV-2 vaccine”] and [“Rheumatic diseases” or “Immune-mediated inflammatory diseases” or “Autoimmune diseases” or “Rheumatic and musculoskeletal diseases”, or “Inflammatory rheumatic disease”] and [“side effects” or “adverse effects” or “safety”]. Based on this search, 24 studies with specific data on side effects, more than 100 study subjects and a publication date from January 1st, 2021 to June 1st, 2022 were included in the review. The studies' corresponding data that were extracted included year of publication, country where the study was conducted, disease types, numbers of patients and controls, vaccination type, data-collection period after the vaccination and side effects. Side effects, including specific incidents and presentations of systemic reactions as well as local reactions, and the incidence and severity of disease flares were recorded.
